# Supplementary material for: The plague of 1720 and migration in Martigues (France) in the 17th and 18th centuries
Source: PLoS One. 2026 Apr 16;21(4):e0346747. doi: 10.1371/journal.pone.0346747 (PMC13086348; doi:10.1371/journal.pone.0346747)
Supplement: S1 File — S1.1. Parish of Jonquières (Municipal Archives of Martigues, GG 26-41), parish of Ferrières (Municipal Archives of Martigues, GG 46-52), parish of l’Île (Municipal Archives of Martigues, GG 11-22), subsidiary parish of La Couronne (Municipal Archives of Martigues, GG 43-45). S1.2. According to the 1716 enumeration conducted at the request of Intendant Cardin-Lebret, entitled État du nombre des familles et des personnes de chaque lieu de Provence, from which only the total population and the number of families for each locality in Provence have survived. This census is archived in the Bibliothèque Nationale under the reference, B.N. ms. fd. fr. 8908 and was partially published by Jean-Noël Biraben (1975, [2, t. 1, Annexe1]. This figure may be lower than the actual number, as it may take into account only the urban population and not that of the surrounding rural area. For comparison, the Dénombrement général et particulier des communautés de la province et intendance de Provence, divisées par vigueries et fait par têtes au mois d’août 1765 records 5,559 inhabitants for Martigues, described as a “community of three parishes: L’Île, Jonquières, and Ferrières,” and 1,519 in the countryside, giving a total of 7,119 inhabitants. To this must be added 100 Provençal foreigners and 39 non-Provençal foreigners. This document is transcribed in volume 5 (page 927) of Abbé Expilly’s Dictionnaire géographique, historique et politique des Gaules et de la France (Paris, 1762-1770, 6 vol. in-fol. Unfinished). S1.3 Municipal Archives of Martigues, CC 390. (DOCX) [file pone.0346747.s001.docx]

**S1. Sources**

S1.1. According to the 1716 enumeration carried out at the request of Intendant Cardin-Lebret, entitled “*État du nombre des familles et des personnes de chaque lieu de Provence*”, from which only the total population and the number of families for each locality in Provence have survived. This census is preserved at the National Library under the reference, B.N. ms. fd. fr. 8908) and was partially published by Jean-Noël Biraben (1975, [2, t. 1, Annexe1].

This figure may be lower than the actual number, as it may take into account only the urban population and not that of the surrounding rural area.

For comparison, the « *Dénombrement général et particulier des communautés de la province et intendance de Provence, divisées par vigueries et fait par têtes au mois d’août 1765 »* records for Martigues, described as a “community of three parishes: L’Île, Jonquières, and Ferrières,” 5,559 inhabitants in the town and 1,519 in the countryside, giving a total of 7,119 inhabitants, to which must be added 100 Provençal foreigners and 39 non-Provençal foreigners. This document is transcribed in volume 5 (page 927) of Expilly’s *Dictionnaire géographique, historique et politique des Gaules et de la France*. (Abbé Expilly. Dictionnaire géographique, historique et politique des Gaules et de la France, Paris, 1762-1770, 6 vol. in-fol. Inachevé)

S1.2. Parish of Jonquières (Municipal Archives of Martigues, GG 32), parish of Ferrières (Municipal Archives of Martigues, GG 47), parish of l’Île (Municipal Archives of Martigues, GG 16).

S1.3 Municipal Archives of Martigues, CC 390.
