# Supplementary material for: The plague of 1720 and migration in Martigues (France) in the 17th and 18th centuries
Source: PLoS One. 2026 Apr 16;21(4):e0346747. doi: 10.1371/journal.pone.0346747 (PMC13086348; doi:10.1371/journal.pone.0346747)
Supplement: S2 Fig — (DOCX) [file pone.0346747.s002.docx]

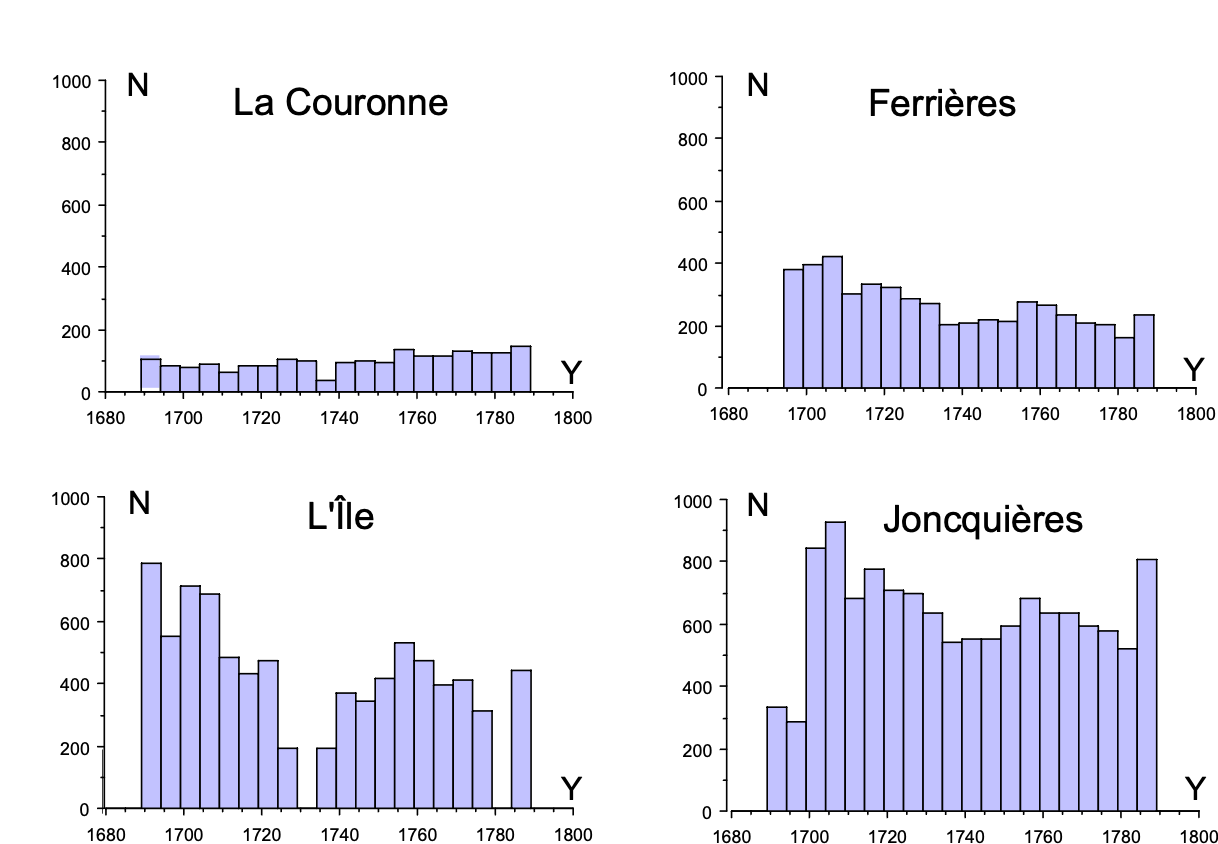


**S2 Fig. Distribution of number N of baptisms by year (Y) in the four districts of Martigues between 1689 and 1789**
