# Supplementary material for: The plague of 1720 and migration in Martigues (France) in the 17th and 18th centuries
Source: PLoS One. 2026 Apr 16;21(4):e0346747. doi: 10.1371/journal.pone.0346747 (PMC13086348; doi:10.1371/journal.pone.0346747)
Supplement: S3 Fig — Y:baptism year. (DOCX) [file pone.0346747.s003.docx]

**S3 Fig. Table of available (1) or missing (0) data by year and district of Martigues. Y: baptism year**
