# Supplementary material for: The plague of 1720 and migration in Martigues (France) in the 17th and 18th centuries
Source: PLoS One. 2026 Apr 16;21(4):e0346747. doi: 10.1371/journal.pone.0346747 (PMC13086348; doi:10.1371/journal.pone.0346747)
Supplement: S4 File — This example illustrates the calculation of the number of occurrences of surnames Abeille and Abille by period and by district, normalized according to the number of years of available observations in each district, and then combined to estimate the number of births per year and per period for Martigues as a whole. N denotes the number of births. The resulting estimates are reported in Table 5 in the main text. (DOCX) [file pone.0346747.s004.docx]

**S4 Example of calculation formula [1]**. Calculation of the number of occurrences of surnames Abeille and Abille by period and by district, normalized according to the number of years of available observations in each district, then combined to produce an estimate of the number of births per year and per period for Martigues as a whole. N represents the number of births. This result is taken from fig 5 in the text.
