# Supplementary material for: The plague of 1720 and migration in Martigues (France) in the 17th and 18th centuries
Source: PLoS One. 2026 Apr 16;21(4):e0346747. doi: 10.1371/journal.pone.0346747 (PMC13086348; doi:10.1371/journal.pone.0346747)
Supplement: S5 Fig — Jenks discretization method (29). Present administrative divisions. Original map created by the authors using the free software Philcarto (http://philcarto.free.fr), with base map © IGN – AdminExpress. (DOCX) [file pone.0346747.s005.docx]

**S5 Fig. Distribution of the number N of births per year (N/year) between 1689 and 1720.** Jenks discretization method (29). Present-day municipal boundaries. Original map created by the authors using the free software philcarto (<http://philcarto.free.fr>) with base map from the public AdminExpress.IGN service (<https://geoservices.ign.fr/adminexpress>)
