# Supplementary material for: The plague of 1720 and migration in Martigues (France) in the 17th and 18th centuries
Source: PLoS One. 2026 Apr 16;21(4):e0346747. doi: 10.1371/journal.pone.0346747 (PMC13086348; doi:10.1371/journal.pone.0346747)
Supplement: S6 File — (DOCX) [file pone.0346747.s006.docx]

**S6** **Lemmatisation.** The first strategy to lemmatization consists in calculating the Levenshtein distance [1].  *L* between each pair of surnames and considering the possibility of lemmatization for names distant by *L*=1 or *L*=2. These names remain numerous. The number of pairs compared giving a Levenshtein distance of *L*=1 is 4,097; those giving a Levenshtein distance of *L*=2 is 19,147. This comparison will nevertheless serve as a decision-making aid in the ultimate process of the choice of lemmatization. Even a small Levenshtein distance between two compared surnames does not guarantee their lineal identity,

The second strategy to lemmatization is based on the idea that a name represented by several births is more likely to be the form of the name to which the other, less numerous, rare or unique (hapax) forms should be lemmatized. Other criteria can be used, such as the location in the surname where the spelling variation occurs, which is more significant at the beginning of the name than at the end. Another criterion is the geographical area where the two forms being compared are attested, one being extremely local and the other rarely found elsewhere.

Take, for example, the forms abeille and abille. Their Levenshtein distance is only *L*=1, but abeille is extremely common while abille can be found just once in Martigues (before 1721) and nowhere else in the reference surname corpus of INSEE (D-insee). This is most certainly a transcription error, in that the surnames of the two other children of the same father are spelled abeille. Lemmatization is justified in this case. The same goes for the five different spellings, malava, malaval, malavale, malavarde and malavart. Only the malaval variant is confirmed in the INSEE file, while the other four variants are confirmed solely for a single birth each, in Martigues and before 1721 only. Lemmatization thus makes sense here, though it would have to be confirmed that these different forms are to be found in the same genealogical line.

However, lemmatizing between cambon and jambon would be ill-advised, since the two forms can be found in the corpus and the variation is at the beginning of the name (see below). Other examples are more delicate, such as molinier and moulinier. Both variants are present in Martigues, before and after 1720, and both can be found widely in Bouches-du-Rhône and in France. Without genealogical verifications, lemmatizing can prove risky.

Lemmatization can be justified based on further considerations. This is the case for farrand and ferrand (and their variants). These two surnames have been passed on from father to son, but farrand is confirmed only before 1720, primarily in the Île district (eight births), and is much rarer than ferrand (39 births), which is present in all districts and in both periods. Is this a single name with two variants? To make sure, we studied the persistence of these names in the Bouches-du-Rhône department after 1789. While ferrand remained extremely common, farrand disappeared entirely. It thus seems reasonable to consider farrand as a simple spelling variation of ferrand, even though farrand continued to exist, but in extremely low numbers (only 9 farrand births between 1891 and 1915 in mainland France, INSEE data). Consequently, farrand and ferrand will be considered here as a single surname.

More complex is the case of ‘soureillet’, to which the following spellings could be related: soubeillet, soulaillet, souleille, souleillet, soulilet, soulillet, soulleillet, soulleyet, soureillet, sourelier, sourlier (among others). Despite the plethora of variants, this is an easier case to rule on, as the sole form truly present in Martigues after 1720 was sour(r)eillet, with 22 births. Only seven and nine of these variants existed in Martigues respectively before and after 1720 (mainly soulaillet, souleillet and soureillet after 1720), just two forms are confirmed between 1789 and 1840 (soulaillet and souleille), and no such forms are confirmed between 1840 and 1915, suggesting the disappearance of lines or a drastic standardization of the spellings of the surname. An equally high number of variants exists for bonin (3 births in P1, 5 in P2), bonnin (0 and 2, respectively), bounan (0 and 1), bonnau, bounin (0 and 18), bounan (0 and 1), bouneu (0 and 4), bounot (5 and 7), bonnet (6 and 24) and bounet (3 and 14). While bonin and bounin are the names of a single family line, how to explain the absence of bounin before 1719 and its predominance after 1720?

It is easier to rule on the case of embrassevin and brassevin. This is most certainly the same surname. For the same couple of parents, the surname of the father, first name Poncet, was given sometimes as embrassevin and others as brassevin (the mother was called maunière Catherine), and their children are registered sometimes as embrassevin and others as brassevin. This configuration is observed for several couples.

Many names have feminized variants, such as martin and martine (one martine in 1722), coulet and coulette, granier and graniere, raynaud and raynaude, violet and violet(t)e, arnaud and arnaude (the last arnaude in 1720). A detailed analysis of these names shows that feminized forms, which were quite common at that time, occur almost exclusively before 1721 and virtually disappear thereafter. It is difficult to determine whether this pattern reflects differences in the sources, an actual abandonment of feminine forms after 1720, or a deliberate ‘defeminization’ introduced by later genealogists. Whatever the cause, we chose to standardize all feminized forms to their masculine equivalents.

The strategy implemented in this study is to lemmatize while at the same time exploring the distributions of the number of baptism names classified in the various categories detailed below. The basic idea is that a name represented by several births is more likely to be the form of the name to which the other, less numerous, rare or unique (hapax) forms should be lemmatized. Whatever the case, the analyses will be carried out before and after lemmatization, and we will show that the major correlation between these two approaches suggests that lemmatization may not be essential in the interpretation of the results of interest to us here.

To meet our objective of reporting on changes in the structure of the population of Martigues resulting from the plague, we will draw on the statistics set out in Table 1 based on pre-lemmatization data. These include the number of surnames absent before and after 1720, as well as the surnames present in 1, 2, 3, between 4 and 10, and more than 10 births.
