# Supplementary material for: The plague of 1720 and migration in Martigues (France) in the 17th and 18th centuries
Source: PLoS One. 2026 Apr 16;21(4):e0346747. doi: 10.1371/journal.pone.0346747 (PMC13086348; doi:10.1371/journal.pone.0346747)
Supplement: Attachment — Submitted filename: PONE-D-25-46489R1_Response to Rev_V2.docx [file pone.0346747.s009.docx]

==========================================================================

==========================================================================

The reviewers' comments are in black, our responses are in blue, and the modified parts of the text (in addition to those already in the text) are in red.

==========================================================================

==========================================================================

Reviewer 4

==========================================================================

- Lines 95–97: The authors state, “We will take account of the fact that Martigues at the time was composed of four districts, La Couronne, Ferrière, L’Île et Jonquières, with different socio-economic profiles.” However, the socio-economic dimension is scarcely developed in the analysis.

We have removed the end of the sentence,

==========================================================================

-Lines 126–144 (esp. 130–134):

The calculations are not fully clear. Please expand this section with a brief, step-by-step explanation (variables, formulas, and any assumptions) so the procedure can be reproduced.

The paragraphs and wording have been modified to make them clearer. An example of calculation is now given as asked by the reviewer, in Supplementary Information S4:

====================,=======================================================

-Line 150: Please indicate, in parentheses, the exact years covered by P1 and P2. This is not immediately obvious (even if inferable from line 131).

It is now given in S4:

==========================================================================

-Lines 253–256: Was the change in surname reporting specific to the study population, or do you observe similar shifts elsewhere in the region during the same period? A brief comparative note or citation would help

The text has been modified I, the supplementary information S7 as:

A detailed analysis of these names shows that feminized forms, which were quite common at that time, occur almost exclusively before 1721 and virtually disappear thereafter. It is difficult to determine whether this pattern reflects differences in the sources, an actual abandonment of feminine forms after 1720, or a deliberate ‘defeminization’ introduced by later genealogists. Whatever the cause, we chose to standardize all feminized forms to their masculine equivalents.

==========================================================================

-Line 426 (wording):“before 1721 but found there after 1720” should read “before 1720 but found there after 1721.”

Changes are done to be clearer :

“Type 2 names are those that do not appear in Martigues before 1721 (1689-1720) but are attested there after 1720 (1721-1789)”

==========================================================================

- able 6: Please define the symbols “m” and “s” in the table caption or a footnote.

m is the mean and s the standard error. Now added in the table caption

==========================================================================

-Related literature: Are you aware of previous studies that use surnames in a similar way to address comparable questions? A short paragraph situating your approach within that literature would strengthen the contribution.

Many studies use surnames to deduce migration patterns over time and space. These are now briefly mentioned in the text, but we are not aware of any other work in which surnames are used in such an epidemiological and historical context as in this study.

===========================================================================

-Lines 664–667: Could the observed fertility decline also reflect population loss due to the plague (e.g., compositional change, marriage-market effects)?

New comments are integrated on this subject, with reference to Biraben and Blum:

“Few historical demographic studies have focused on Provence in the eighteenth century. The works of Louis Henry (1978) and Alain Blum (1988) reveal pronounced regional differences in fertility patterns from the first half of the eighteenth century onward, with the southeastern France exhibiting lower final fertility rates than northern and western regions. In Martigues, these fertility trends were likely amplified by the severe population decline. After the plague, no rapid or sustained demographic recovery occurred, largely due to the stronger pull exerted by the nearby city of Marseille.”

===========================================================================

REVIEWER 1

*First issue :*

- Abstract and introduction should better highlight specific, interesting and new findings from the authors’ quantitative analysis.

The abstract has been enhanced to include the main general conclusions

The surname method used here indicates that the plague caused an exceptionally large renewal of approximately 50% of the stock of surnames, and thus of the population bearing those names. It also shows that fertility declined significantly among individuals whose surnames were already present among those who died of the plague. Finally, the results demonstrate that population renewal was achieved primarily through immigration, mainly from neighbouring municipalities.

The current introduction makes quite general points about the usefulness of names-based analyses but does not clearly communicate what we learn from the application of these methods and sources to the particular 1720s Martigues outbreak. To be sure, some general conclusions are drawn in section 4 (“Discussion”), but I would like to see them highlighted from the beginning on. In short, I recommend that the authors give a clear idea, based on quantitative evidence, as to

(1) “how important” out-migration, mortality and subsequent in-migration were for Martigue’s 1720 plague incident,

(2) in how far their conclusion deviate or extend upon existing (qualitative) evidence on the 1720 plague, and

(3) whether we would profit from attempting similar analyses for other epidemic outbreaks.
- Estimation of name frequencies (page 6):

Thanks to the reviewer ‘remarks, the introduction has been extensively revised. It now includes references that address the relationship between surnames, migration, and fertility. Readers can refer to them. In response to the reviewer, we also have added further details on the situation in Martigues at that time, on the nature of the plague, and on the conditions surrounding its outbreak in Martigues. Furthermore, we believe it would be inappropriate to anticipate a presentation of our results in the introduction when they are discussed further on. In response to the reviewer, we could point to the change now made to the abstract, which briefly presents the key results.

We now emphasize the pioneering nature of this study and the fact that it is quite unique due to the exceptional preservation of the Martigues archives; it cannot be replicated as such in other cities in Provence, or even elsewhere in France, as few have preserved lists of victims' names and a complete and comprehensive record of civil status documents at the city level.

===========================================================================

*Second issue*

- Estimation of name frequencies (page 6): The authors present a pragmatic way to estimate true name frequency by period given that data for some year-region cells is missing in the original sources. However, from the description and formula given in the paper, it is unclear to me why the four different districts are not weighted by population size (or the number of births) when computing Martigues-wide surname indices. This seems sensible given that the 4 districts seem to vary strongly in population/birth number sizes (figure S3). If the authors think that such weighting is unnecessary in the aggregation, this choice should be explained.

This point raises the question of whether to use the number *n_s_* of people with a given surname *s* in a given district or to analyze its frequency *n_s_/N*, where *N* is the total number of people in that district. We did not choose this last alternative. The first reason is that it would not change the typology of names (type 1, type 2, or type 3) on which the method is based. The second reason is that we are looking for a total estimate of the *number* of people with a given name in Martigues as a whole and not an estimate of the *frequency* of name per district. If the surname data were expressed in frequency per district, this would amount to adding up the four frequencies of the name in order to estimate its frequency in Martigues as a whole, which would not be right and would obscure the demographic fact that some districts had a larger population than others, which must be considered. The correction to consider the different number of years of observation per district remains still necessary so that each district has the same number of years of observation, and they can be summed to provide a value for Martigues as a whole.

The paragraph describing calculations has been modified to make it clearer. An example of calculation is also given as asked by the reviewer in Supplementary information S4.

===========================================================================

*Third issue*

Interpretation of name-frequency changes as indicator for population turnover: It seems plausible to me that certain surnames would always be more likely to spontaneously reappear in a population, even if their original carriers all disappeared due to a shock (i.e. died or emigrated). I am thinking of surnames that relate to common occupations, places, resources or physical properties of the area. The authors’ current approach does not seem to account for this issue and rather conceptualizes the pool of possible names as static/given. If this is a defensible/useful assumption in the authors’ view, I would like to see a comment on this issue.

We add theses part in the introduction:

The example presented here focuses specifically on the local demographic consequences of the last major plague epidemic in France, during the early modern period, using an innovative surname-based approach. This method is suitable because, by the eighteenth century, surnames were already passed down from father to children, albeit sometimes with minor variations in spelling. No surname could appear spontaneously, as every individual bore a baptismal name, even those who came from outside the community. Consequently, analyzing changes in a population’s stock of surnames provides a reliable indicator of either extinction, disappearance, or migration. Previous studies have demonstrated the effectiveness of this method in various contexts and periods, both past and present

===========================================================================

*Forth issue*

- Source of population inflow (line 710ff.): The authors claim that population inflow from surrounding municipalities could have accounted for a substantial part of the population replenishing after the plague shocks. I do not understand how this fits together with the earlier statement that many municipalities in the region were hit by the plague (not only Martigues), and 1/3 of the regional population died. Didn’t other towns in the region face similar population losses, and wouldn’t this undermine their ability to provide Martigues with new in-migrants?

Actually, although the towns surrounding Martigues were also affected by the plague, this did not prevent their inhabitants from migrating around (as people of Martigues did) and therefore migrating toward Martigues. There would only be a contradiction if these neighboring towns had escaped emigration, which is unrealistic.

===========================================================================

*Fifth issue*

Exploring the correlation between name “types” and occupations: The original data used by the authors contains rich data on the occupations of inhabitants and the authors occasionally mention interesting concentrations of particular occupations among specific surnames or “types”. However, there is no attempt at a more formal analysis. I would like to see whether, statistically, surnames with an above-average representation in the three main socioeconomic classes in Martigues (seafaring/sea-related occupations, merchants, others such as agriculture) were more likely to disappear, newly appear, or stay stable between the two periods. This seems like an interesting and straight-forward extension to the paper.

Our genealogical data are taken from baptism records, which, sometimes, mention the fathers' professions, sometimes not. The reviewer 's idea could certainly be developed further, but this would not be so simple given the wide variety of occupations and how they evolved over the course of the XVIII°century. Furthermore, literature tells us that the plague affected all social classes and all professions indiscriminately. So, this could be the subject of another study.

===========================================================================

There are some minor issues with the draft:
- Line 51: The statement that 242 communes in France were affected by the plague shock is not useful without an idea how many communes there were in total.

Now we add in the introduction:

Despite quarantines—undoubtedly implemented too late—it gained momentum in the surrounding villages, spreading across Provence and into Lower Languedoc and the Comtat Venaissin. The epidemic’s toll varied sharply from one community to another: some were spared completely, while others suffered severe losses, such as La Valette, with 1,068 deaths out of 1,600 inhabitants [12]. Urban centres were the most severely affected. ===========================================================================

Moreover, given the highly approximative death count (“nearly 120k”), it seems implausible that the count of affected communes is certain. I would omit this information and focus on the estimated death count in the region.

120k deaths from the plague is not so inaccurate given that epidemiological records in the 18th century don’t allow for the same level of accuracy as today...This number was estimated following extensive research by historians, now referenced in the text, who did their best with the available sources.

===========================================================================

- Line 59: The sentence “See also the location of Marseilles” seems interrupted/incomplete (?).

OK

- Line 86: Citation [7] does not seem to point to the right object in the bibliography.

OK

===========================================================================

- Line 101: Please clarify whether missing data is completely clustered by year (i.e. if a year is not missing, all baptisms in that year are transcribed). This seems to be assumed in your estimation, thus I would like to clarify.

The parish registers of Martigues offer an unbroken record extending over more than two centuries. However, not all transcriptions of the data were available at the time of this study. Few years was not yet recorded, and the births of these years are hence absent.

===========================================================================

- Line 109: What is a “significant” surname?

Some of the old parish records were not allowing to identify the name of baptism, either because it was absent, or because the writing was too unclear to link it to a known name. So, the name was unusable. Text is modified to be more accurate

- Line 188: characters “1” and “[“ mixed up.

OK, done

- Lines 312/313: You are referring to names with more than 3 baptisms, so the term “over-four” is misleading.

OK, done

- Line 516: Character “]” missing.

OK

===========================================================================

- Line 666: You speculate that the remarkable fall in fertility after the plague “cannot necessarily be attributed to the epidemic alone, though it may have played a role in the trend.” At this point it is unclear to me on which basis you can make that claim. Starting in line 689, you then pick up this thought again, and seem to conclude that the plague indeed changed fertility behavior substantially? I recommend slightly restructuring/rewriting this part to minimize the need for interpretation on part of the reader.

We think that we are not so affirmative. Anyhow, we introduce a new paragraph about fertility in the XVIII°.

Few historical demographic studies have focused on Provence in the eighteenth century. The works of Louis Henry [30] and Alain Blum [31] reveal marked regional differences in fertility patterns from the first half of the eighteenth century onwards, with the southeast showing lower completed fertility rates than northern and western France. In Martigues, fertility trends were likely intensified by the drastic decline in population. Following the plague, demographic recovery was neither rapid nor sustained, largely due to the stronger attraction of the neighbouring city of Marseille.

===========================================================================

Line 689 is modified to be more precise:

“The results clearly indicate a substantial renewal of the population after 1720, approximately 50%, which may be attributable to the effects of the plague.”

===========================================================================

- Figure S5 (appendix) shows many municipalities with 0 births per year between 1689 and 1720. Are these uninhabited? Seems implausible.

Parish registers are not available for the moment for these municipalities

===========================================================================

- The language is occasionally vague and colloquial (e.g. line 253: “(please excuse us for this g endered bias…)”).

We have totally changed this part in the supplementary information S6:

“A detailed analysis of these names shows that feminized forms, which were fairly common at that time, appear almost exclusively before 1721 and then almost systematically disappear. It is difficult to determine whether this pattern reflects differences in sources, an actual abandonment of feminine forms after 1720, or a deliberate defeminization introduced by genealogists working on later records. Whatever the underlying cause, we choose to convert feminine forms into their corresponding masculine forms..”

===========================================================================

===========================================================================

REVIEWER 2

My largest concern is the authors’ insufficiently critical discussion of the parish register data and their well-known limitations. Usually, parish registers from the early 18th century do not provide reliable information on the number of individuals present at a given moment in the parish. Individuals only appear in the data if they exhibit vital events like marriage, childbirth, and death. Labor migrants and other population groups are often recorded poorly or not at all. The estimation of the ‘population at risk’ is therefore biased and research questions addressing general trends of the study population must be assessed very carefully. I encourage the authors to think about the potential biases of using parish registers as a source

We are aware that parish records do not cover the entire population. In the 18th century, data covering exhaustively the entire inhabitants living in a parish or an area are rare and/or difficult to collate. That why we do only use the baptisms as a source and their list is quite exhaustive. So our conclusions concern only the evolution on time of this restricted population of newborns (and their surnames) in a given place. We also mention the exceptional quality of the baptismal records in Martigues in the introduction.

==================================================================

The authors mention on page 3 line 64 that there were 5,886 individuals living in Martigues in 1716. (Did this number derive from a census?) If their parish register data allows the reconstruction of life courses, it would be interesting to see how many individuals were present in Martigues in the same year according to the parish register data.

This clarification is provided in footnote S1.1 in the revised introduction.

According to a regional census, entitled “*Etat du nombre des familles et des personnes de chaque lieu de Provence*” (preserved at the National Library under the reference, B.N. ms. fd. fr. 8908) and published by JN Biraben (1975, t. 1, Annexe1), 5,886 individuals were living in Martigues in 1716.

==================================================================

Another major flaw is the framing of the paper. It is unclear to me what the aim of the paper is. So far, the reader is educated in detail about the methods used, e.g. lemmatization techniques, and the results, changes in the surname distribution before and after the plague and the grouping into categories. But what do we learn from this? It remains unclear whether the authors want to test their methods or whether they have used the methods to investigate the change of the population structure in Martigues. The title suggests the latter, but for this enterprise the paper must focus and refer to other studies that have investigated population change in responses to disasters.

Our method based on surnames, which is fairly simple but original compared to traditional surname-based methods, has been implemented solely to attempt to answer the question posed: To what extent does an event such as the plague alter the structure of a population?

On the last page of discussion, there are some speculations on the mechanisms of population change structure (plague mortality differentials between families, selective migration). Further, the whole paper lists only 14 references which only refer to data and methods issues. If the focus of the paper lies on the authors’ technique, then the framing and the title must be revised. It

We have added the few references available concerning the demographic situation in southeastern France at the time in question. Introduction and discussion have been modified to introduce them.

===========================================================================

INSEE

OK, done

===========================================================================

===========================================================================

**Reviewer 3**

I would have expected to find some references to historical/historical-demographical/demographic studies into the effects of major disruptive events such as wars and epidemics (Alfani on the plague for example, and/or Scheidel with The Great Leveler). It is important to add a bit of literature, to acknowledge the research that has been done so far, and to be able to put this finding into perspective.

We have now added such references

===============================================================

I would advise the authors to restructure the article and focus more on the main findings and trends, with fewer highly specific examples, details and exceptions.

We now reject the details of the lemmatization in the supplementary information (S7).

===============================================================

For starters, the examples given from line 201 until 247 are a bit much. It would be better to provide an overview of how many names were difficult to lemmatize, and what percentage was easy, and sufficing with only one or two examples. The presentation of all these examples may appear thorough, but it is not clear to the reader whether this list is exhaustive.

We now reject the details of the lemmatization in the supplementary information (S7).

===========================================================================

It is also not necessary to provide an exhaustive list, since the examples allow the reader to get an idea, and the results will later be given before and after lemmatization.

Moreover, tables 2, 4 and 5 are very elaborate, which hinders the quick conveyance of the main message to the reader. Could each table be moved to an appendix, and be replaced by more summarizing tables in the main text? I let it to the discretion of the authors to decide how to summarize and what message they want to convey, but a suggestion could be to summarize the cases by the Plague variable (where a p refers to plague deaths and an n to no plague deaths?

Where should cuts be made? We believe that the details (lemmatization and statistics associated with each name) constitute a large part of what makes this work, difficult, interesting and original. It would be possible to include in the table only names which are mentioned in the text. However, we believe it is important to be able to evaluate names in relation to each other. We therefore wish to retain the tables as they are.

===============================================================

It may also be wise to order the table (the one that may be moved to an appendix) alphabetically (or by any other systematic approach, but I couldn’t find a system at the moment), to make it easier for your reader to digest.

The table are already ordered, not alphabetically, but by frequency of name by year (column N_1 total/Year (Table 1) and N_2 total/year (Table 4) and by ratio of N_2 (table 5).

===============================================================

Table 6: it is not clear to me what m and s refer to, could the authors specify? Overall, it may be helpful to include more informative headings in the tables, so they need less going back-and-forth between the explanatory text and the table.

We increase the information in the headings

===============================================================

The discussion, however, fails to answer the initial objective listed in line 84-87 of studying whether some families were impacted more than others, based on socioeconomic characteristics. It is only mentioned in lines 704-706 that the occupation of the father could be an explanatory behaviour for migration, but a systematic analysis of socioeconomic background is lacking. I would advise the authors to either rephrase the aim of their study, or to include a systematic analysis of socioeconomic background (which might be a bit complex, but which would really add to the study!)

We exclude socio-economic discussion, since we know that the plague struck all social classes indiscriminately.

===============================================================
